# Determining modular organization of protein interaction networks by maximizing modularity density

**DOI:** 10.1186/1752-0509-4-S2-S10

**Published:** 2010-09-13

**Authors:** Shihua Zhang, Xue-Mei Ning, Chris Ding, Xiang-Sun Zhang

**Affiliations:** 1 Academy of Mathematics and Systems Science, Chinese Academy of Sciences, Beijing 100190, China; 2 Key Laboratory of Random Complex Structures and Data Science, Academy of Mathematics and Systems Science, Chinese Academy of Sciences, Beijing, China; 3 College of Science, Beijing Forestry University, Beijing 100083, China; 4 Department of Computer Science and Engineering, University of Texas at Arlington Arlington, TX 76019, USA

## Abstract

**Background:**

With ever increasing amount of available data on biological networks, modeling and understanding the structure of these large networks is an important problem with profound biological implications. Cellular functions and biochemical events are coordinately carried out by groups of proteins interacting each other in biological modules. Identifying of such modules in protein interaction networks is very important for understanding the structure and function of these fundamental cellular networks. Therefore, developing an effective computational method to uncover biological modules should be highly challenging and indispensable.

**Results:**

The purpose of this study is to introduce a new quantitative measure modularity density into the field of biomolecular networks and develop new algorithms for detecting functional modules in protein-protein interaction (PPI) networks. Specifically, we adopt the simulated annealing (SA) to maximize the modularity density and evaluate its efficiency on simulated networks. In order to address the computational complexity of SA procedure, we devise a spectral method for optimizing the index and apply it to a yeast PPI network.

**Conclusions:**

Our analysis of detected modules by the present method suggests that most of these modules have well biological significance in context of protein complexes. Comparison with the MCL and the modularity based methods shows the efficiency of our method.

## Background

Understanding the cell as a system of interacting components is a fundamental goal of current biology. Various types of biological networks are being constructed in cellular systems including PPI networks, gene regulatory networks and metabolic networks, etc. Exploring how molecules interact to form cellular machinery is a key task in systems biology. The well-understood graph-theoretical concepts has become a powerful tool to explore the topology, organization, function and evolution of biological networks. In this field, recent studies have made great progresses which considerably expanded our insights in the organizational principles and cellular mechanisms of cellular systems [1,2].

Modularity has been considered to be one of the main organization principles of biological networks in the past decade years. Biological modules as a critical level of biological hierarchy and relatively independent units play special roles in biological systems [1]. How to uncover modular structures in various biological networks is a basic step for understanding cellular functions and organizational mechanisms of biosystems. For example, by using the network partition, Zhao *et al*. (2006) investigated the functional and evolutionary modularity of human metabolic networks from a topological perspective [3].

In the past few years, a huge number of computational methods have been developed for detecting network modules and analyzing the network structure of biological networks. Hierarchical clustering has been proven to be useful tools for analyzing biological networks. Ravasz *et al*. (2002) studied the hierarchical modular organization of metabolic networks based on a topological linkage matrix. Researchers of three groups [4-6] employed hierarchical clustering based on three different clustering methods respectively to analyze the modular structure of yeast protein interaction networks. The diffusion kernel of graph was also suggested as a universal similarity metric to construct the clustering tree of networks [7]. However, this type of approaches may generate many identical distances (similarity) and leads to the ‘tie in proximity' problem during hierarchical clustering. This type of method imposes a stringent tree structure on the network which is highly sensitive to the metric used to assess (dis)similarity, and typically requires subjective evaluation to define modules. And the evaluation of these (dis)similarity measures for hierarchical clustering is not an easy problem.

Several studies of protein interaction networks have focused on detecting highly connected protein modules [8-10] which generally correspond to meaningful biological units such as protein complexes and functional modules. In general, these approaches only employ local connectivity among proteins and neglect many peripheral proteins that connect to the core protein clusters with few links. However, biological networks including PPI networks are generally very sparse. Most methods only identify strongly connected subgraphs as modules, so only a few modules were detected [9,11,12]. And biologically meaningful sparse protein modules are ignored by these approaches and those lost peripheral proteins may represent experimentally true interactions. Furthermore, because these approaches heavily rely on the local topological connectivity, they ignore the impact of global organization of networks. But biological networks are globally coordinated system, so the local connectivity based methods can not be employed to explore the relationship among modules. Another important factor is the noise of interaction data, other sources such as the function annotation data and gene expression data have been integrated into protein interaction networks to improve the effectiveness of module detection [13,14].

One popular class of methods for dissecting modular structure in the field of general complex networks is based on optimizing a global quality function called modularity [15,16] to partition the network into modules. And it has been comprehensively adopted to analyze biological networks [3,17-19]. However, it has recently been shown that the resolution of the modularity based methods is intrinsically limited. It fails to find small communities in large networks—instead, groups of small communities turn out merged as larger ones [20]. Li *et al*. (2008) proposed a novel quality function called modularity density (*D*) which aims to conquer the resolution limit problem in modularity [21]. They have tested it on many kinds of small networks for illustration but not on large real networks.

In this study, we aim to introduce the new quantitative measure modularity density into the modular analysis of biomolecular networks and develop new algorithms for detecting functional modules in protein-protein interaction (PPI) networks. We first adopt the simulated annealing (SA) technique to maximize the modularity density and evaluate its advantages on a suit of simulated networks where the modules are known. In order to conquer the computational burden of SA procedure, we adopt a spectral fc-means method for optimizing the measure and apply it to a yeast PPI network. Our biological analysis of detected modules suggests that most of these modules carry distinguished biological significance. We also make a comparison of our method with other two methods including the popular MCL and modularity based methods to verify its effectiveness.

## Materials and methods

### Definition of modularity and modularity density

The popular modularity *Q* is defined by Newman and Girvan (2004). Briefly, when the nodes of a network are divided into modules, one can compute it as follows:

where *m* is the number of modules, *L* is the total number of edges in the network, *l_i_* is the number of edges between nodes in module *i*, and *d_i_* is the total number of degrees of the nodes in module *i*. The highest *Q* value of all possible module separations is called the network modularity. In the past studies, empirical and simulation studies showed that the network partition method of maximizing modularity *Q* (MQ) has good performance. However, Fortunato and Barthelemy (2007) recently pointed out the serious resolution limits of this method, and claimed that the size of a detected module depends on the size of the whole network. The main reason is that the modularity *Q* does not capture the information of the number of nodes in a module, and the choice of partition is highly sensitive to the total number of links in the network.

In the following, we introduce the so-called modularity density *D* which was proposed as an alternative measure for describing the modular organization [21]. The characteristic of this measure is that it is related to the density of subgraphs. We first define the average modularity degree of subgraph *G_i_* (*V_i_, E_i_*) as follows:

where *aid* (*G_i_*) is the average inner degree of the subgraph *G_i_*, which equals to twice the number of edges in subgraph *G_i_* divided by the number *n_i_* of nodes in this subgraph. *aod* (*G_i_*) is the average outer degree of the subgraph *G_i_*, which equals to the number of edges with one node in the subgraph and the other node outside it divided by the number *n_i_* of nodes in the subgraph. The intuitive idea is that *ad* (*G_i_*) should be as large as possible for a valid ‘module'. Then the modularity density *D* of a partition *G*_1,_ …, *G_m_* is defined as the sum of all average modularity degree of *G_i_* for *i* = 1, …, *m*. In contrast to *Q, D* can be calculated as follows:

This measure provides a way to determine if a certain mesoscopic description of the graph is accurate in terms of modules. The larger the value of *D*, the more accurate a partition is. So the community detection problem can be viewed as a problem of finding a partition of a network such that its modularity density *D* is maximized. The search for optimal modularity density *D* is a challenging problem due to the fact that the space of possible partitions grows faster than any power of system size.

Moreover, the phenomenon of multiple resolutions or/and hierarchy of modular structures have been observed in biological networks [22]. The modularity density *D* can be extended for this more general case using a tuning parameter λ as follows [21]:

where λ is a value ranging from 0 to 1, and when λ = 0.5, the *D*_0.5_ corresponds to modularity density *D*. By varying λ, we can detect detailed and hierarchical organization of biological systems. In other words, we can divide the network into large modules and small modules using a small λ and a large λ respectively.

### Simulated annealing for maximizing *D* (MD)

In principle, the goal of a module detection is to find the ‘optimal’ partition with largest modularity *Q* or modularity density *D*. Several methods have been proposed for optimizing *Q*. Most of them rely on heuristic procedures or approximate strategies. Here, we employ the simulated annealing (SA) technique to maximize *Q* and *D* to obtain the ‘best’ determination of the modules of a network for evaluating. Simulated annealing is a kind of stochastic search technique for optimization problems. It enables one to find ‘low cost' configurations without getting trapped in ‘high cost' local minima and has many applications in combinatorial optimization problems. In the searching process, a global parameter *T* representing temperature is introduced. When *T* is high, the system can explore configurations of high cost while at low *T* the system only explores low cost regions. Along with the decrease of *T*, ‘low cost' configurations can be reached step by step by overcoming small cost barriers. When identifying modules, the objective is to maximize the quantitative indexes (i.e. *Q* or *D*), thus, the cost is *C* = −*Q* or −*D*. At each temperature, we perform a number of random updates and accept them with probability:

(1)

where *C_i_* (*C_f_*) is the cost before(after) the update.

Specific implementation detail can be seen in [17]. Note that we add a decision clause to ensure that each potential ‘module' is connected. The one that performs best consists in isolating the module from the rest of the network, and performing a nested' SA, entirely independent of the ‘global’ one. In using *Q* and *D* as fitness functions', the method is more direct than those relying on heuristic procedures. Moreover, SA enables us to carry out an exhaustive search and to minimize the problem of finding sub-optimal partitions. We should note that the SA method can't scale to very large networks, but it is an efficient evaluation method for its exhaustive characteristic. Several efficient methods for optimizing *Q* have been proposed, but designing efficient algorithms for optimizing the new measure (*D*) is still an essential and challenging problem.

### Spectral method for maximizing *D* (SpeMD)

Given a network *G* = (*V*, *E*), and denote its vertex set as *V*, edge set as *E* and adjacency matrix as *A*. Given a *m*-partition *P_m_*, define a corresponding *n* × *m* assignment matrix *X* = [*h*_1_,*h*_2_, …, *h_m_*] with *h_ic_* = 1 if *v_i_* ∊ *V_c_*, and *h_ic_* = 0 otherwise, for 1 ≤ *c* ≤ *m*. Observe that since each vertex can only be in one cluster, *X* 1*_m_* = 1*_n_*. We can reformulate *D* in terms of the assignment matrix *X* as follows:

where *B* is the degree matrix. Let  note that  or  then, we can obtain

So the problem of maximizing *D* can then be expressed as:

(2)

From the standard result in linear algebra, the optimal  of the above trace maximization has close relationship with the leading *k* eigenvectors of 2*A* − *B* by relaxing  as an arbitrary orthonormal matrix [23]. We can adopt the corresponding spectral algorithms and use the leading *k* eigenvectors of 2*A* − *B* to optimize the modularity density *D*. To obtain the final network partition, we apply the *k*-means clustering method to cluster eigenvectors. Importantly, the same principle can be derived for *D*_λ_.

### The procedure of the algorithm

Given an upper bound *K* of the number of modules and the adjacency matrix *A* = (*a_ĳ_*)*_n×n_* of a network. The procedure of the algorithm is stated straightforward as follows:

•	Spectral mapping:

1. Compute the diagonal matrix *B* = (*d_ii_*), where .

2. Form the eigenvector matrix *U_k_* = [**u_1_, u_2_, …, u_K_**], corresponding to the *K* largest eigenvalues of 2*A* − *B*.

•	*k*-means: for each value of *k*, 2 ≤ *k* ≤ *K*

1. Form the matrix *U_k_* = [**u_2_, u_3_, …, u_k_**] from the matrix *U_k_* .

2. Normalize the rows of *U_k_* to unit length using Euclidean distance norm: .

3. Treat the rows of *U_k_* as points in *R^k^* and cluster them into *k* clusters using *k*-means or even other clustering methods.

•	Maximizing modularity density *D* or *D*_λ_ with given λ: Pick the* k* and the corresponding partition *P_k_* that maximizes *D* or *D*_λ_.

We should note that this type of spectral clustering technique has been successfully applied to general clustering problems as well as graph clustering problems [24,25]. Here, we explore the characteristic of modularity density *D*, and derive a new spectral clustering based method for maximize *D* (*D*_λ_) (SpeMD). And the SpeMD procedure described here can be seen as a particular manner of employing the standard *k*-means algorithm on the elements of the leading *k* eigenvectors to extract *k* clusters simultaneously. Convergence and computational complexity of the SpeMD procedure are key problems when this method is applied to large complex networks. Fortunately, several strategies can be employed to improve these problems. First, we can initialize the *k*-means such that the starting centroids are chosen to be as orthogonal as possible [26]. This strategy does not change the time complexity, but can improve the quality of convergence, thus at the same time reduce the need for restarting the random initialization process. Second, several fast techniques for solving eigen system have been developed and several methods of *k*-means acceleration can also be found in the literature. Based on this type of techniques, for large sparse networks with *m* ~ *n*, and *k* ≪ *n*, the SpeMD procedure will scale roughly linearly as a function of the number of nodes *n *[25]. Here we didn't consider these ameliorative techniques and only focus on the validity of the SpeMD method.

### Performance measures

The biological significance of the numerically computed modules can be validated by comparing the experimentally determined complexes in the MIPS database [27] with the computed modules. We use a best-matching criteria which was first introduced in [8] to match these two type of protein sets. By minimizing the probability *P_ol_* of a random overlap between a computational protein module and an experimental complex using hypergeometric distribution, we determine the best-matching experimental complex for a protein module. The *P_ol_* is defined as follows:

where *N* is the size of the PPI network, *k* is the number of their common proteins, and |*C*|, |*M*| are the sizes of an experimental complex and a computed protein module respectively.

Furthermore, the geometric accuracy and separation described in the study of Brohee and van Helden [28] are employed to evaluate the performance of the module-detection methods. We first build a contingency table *T*, where row *i* corresponds to the *i* th experimental complex and column *j* to the *j* th module and the value of a cell *T_ĳ_* indicates the number of proteins found in common between complex *i* and module *j*. The contingency table has *n* rows (complexes) and m columns (modules). Using this table, each module partitioning result is compared with the experimental complexes.

**Accuracy:** The complex-wise sensitivity is defined as the maximal fraction of proteins in complex *i* that could be found in one module by the formula:

where *N_i_* is the number of proteins belonging to complex *i*. To characterize the general sensitivity of a partitioning result, a clustering-wise sensitivity is defined as the weighted average of over all complexes by the formula:

Moreover, the cluster-wise positive predictive value  is calculated as the maximal fraction of proteins in module *j* found in the best-matching complex by the formula:

To determine the general PPV (positive predictive value) of a partitioning result as a whole, the clustering-wise PPV is computed as the weighted average of  over all modules by:

Finally, the geometric accuracy (ACC) indicating the tradeoff between sensitivity and predictive value can be obtained by computing the geometric mean of the SN and the PPV as follows:

**Separation:** From the contingency table, the relative frequencies with respect to the marginal sums can be defined for each row and each column as:

Then the separation is computed as the product of column-wise and row-wise frequencies by:

The complex-wise separation  (cluster-wise separation for module ) is calculated as the sum of separation values for a given complex *i* (module *j*) by:

The complex-wise *SEP_co_* and clustering-wise *SEP_cl_* values are computed as the average of  over all complexes, and of  over all modules, respectively:

The the geometric separation (SEP) is defined as the geometric mean of *SEP_co_* and *SEP_cl_* by:

## Results

In this section, we apply the present method to a suit of simulated networks and a yeast PPI network to test its efficiency. We first present detailed numerical results to show the difference of network partition determined by maximizing the modularity density *D* and modularity *Q* with simulated annealing (SA) technique. In general, maximizing *D* (MD) can give more detailed and valid results, while maximizing *Q* (MQ) encounters serious resolution limit in simulated networks.

Then we apply the new spectral method for maximizing the generalized *D_λ_* (SpeMD) to a yeast PPI network to identify functional modules which show significant biological relevance. Comparison with MQ and MCL, we show that the SpeMD can obtain competitive performance with the well-known MCL method and resolve much finer modular structure than MQ method. To extract appropriate modules, the SpeMD and MCL both rely on one parameter. Here, we perform the SpeMD and MCL with adjusted parameters to obtain the ‘best’ geometric accuracy and separation. For SpeMD, we tune *λ* from 0.4 to 0.7 in step of 0.05, and for MCL, we sample inflation parameter values from 1.5 to 2 in steps of 0.1.

### Simulated networks

First we do the comprehensive tests on a group of simulated networks which take on significant modular characteristics. In the work of [21], *D*-based method has been showed to be able to obtain competitive performance with *Q*-based method. However, the size of artificial networks generated by using Newman's popular procedure as well as its variant are too small to show the serious resolution limit problem of *Q*. Therefore, we devise a new type of artificial networks. The network is composed of 2*m* cliques (*mn*_1_-clique and *mn*_2_-clique), and external edges are placed randomly with a fixed expectation values so as to keep the average edge connections *k_out_* of each node to nodes of other cliques. So each network has *m*(*n*_1_ + *n*_2_) nodes and about *m*(*n*_1_(*n*_1_ − 1)/2 + *n*_2_(*n*_2_ − 1)/2) + *m*(*n*_1_ + *n*_2_)*k_out_* /2 edges. In the following test, we let *n*_1_ = 10 and *n*_2_ = 15. Note that we can also relax cliques as dense modules for testing, but here we just show the clique case for convenience.

**Figure 1:**
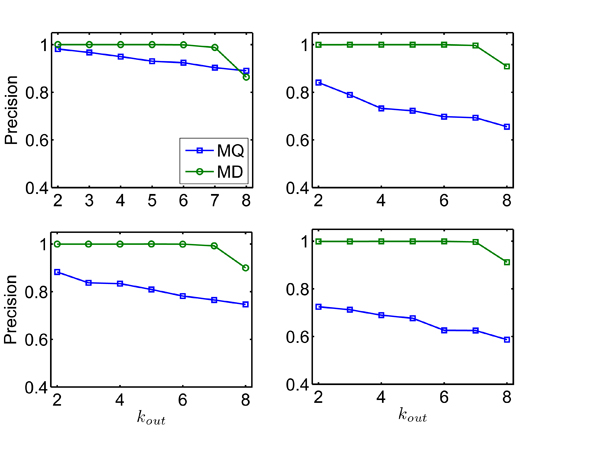
**Community structures of MD and MQ on simulated networks** Comparative test of MD and MQ on simulated networks with known community structures. It is a plot of the fraction of nodes correctly classified with respect to *k_out_*. Each point is an average over 50 realizations of the networks.

**Figure 2:**
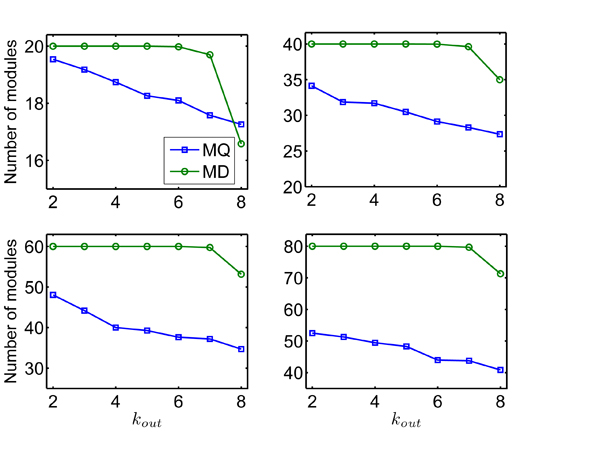
**Number of modules detected by MD and MQ** with the real number of cliques (NC), averaged over 50 network realizations.

The computational results for this experiment are summarized in Figure 1 and Figure 2, where NC is the number of cliques, i.e., *NC* = 2*m*. Figure 1 shows the fraction of nodes that are correctly classified into the communities (Precision) with respect to *k_out_* by MD and MQ respectively. We can see that MD method based on *D*-value performs much better than MQ method under all the different NC. For instance, for 50 random networks with *NC* = 60 and *k_out_* = 5, on an average 99.97% nodes are classified correctly by MD, while only about 72.23% nodes by the MQ. When *k_out_* = 8 which indicates the corresponding networks are difficult to be partitioned, MD still has very high accuracy (>86%).

The most interesting observation is that performance of MD is almost the same, while that of MQ is greatly decreasing with the increase of NC (also the size of networks). For example, for 50 random networks with *k_out_* = 6, always on an average >99.9% nodes are classified correctly by MD on four different sizes of networks with *NC* = 20, 40, 60, 80, while about 92.40%, 78.18%, 69.75%, 62.59% nodes by the MQ respectively. This fact shows the serious resolution limit problem of modularity *Q*, while that can not be observed on the small networks such as the simulated networks using Newman's method.

To test the performance of MD and MQ in selecting the number of communities, we calculate the number of modules. Figure 2 shows the averaged number of modules on four different sizes of networks (*NC* = 20, 40, 60, 80) with respect to *k_out_* by MD and MQ respectively. We can see that MD performs much better than MQ. The MD can almost always identify the right number of modules in four different sizes of networks with *k_out_* ≤ 7. While MQ can not do that. For example, for 50 random networks with *NC* = 60 and *k_out_* = 7, on an average 59.7 modules are identified by MD, while only about 37.20 modules by the MQ. For the harder case (*k_out_* = 8), MD can still do much better than MQ. Actually, even for the easiest case *k_out_* = 2, MQ can not identify the right modules with 52.50 modules for *NC*=80. This uncovers the underlying resolution limit just as pointed in [20]. In summary, the MD can recover the underlying community structure more often than the MQ by a sizable margin in the simulated modular networks. The modularity density *D* more relies on local connectivity of a network and can uncover finer modular structure. While modularity *Q* more relies on size and total links of a network and can lead to serious resolution limit. Moreover, the limit is more serious as size of networks increasing.

### Results on a PPI network

**Figure 3:**
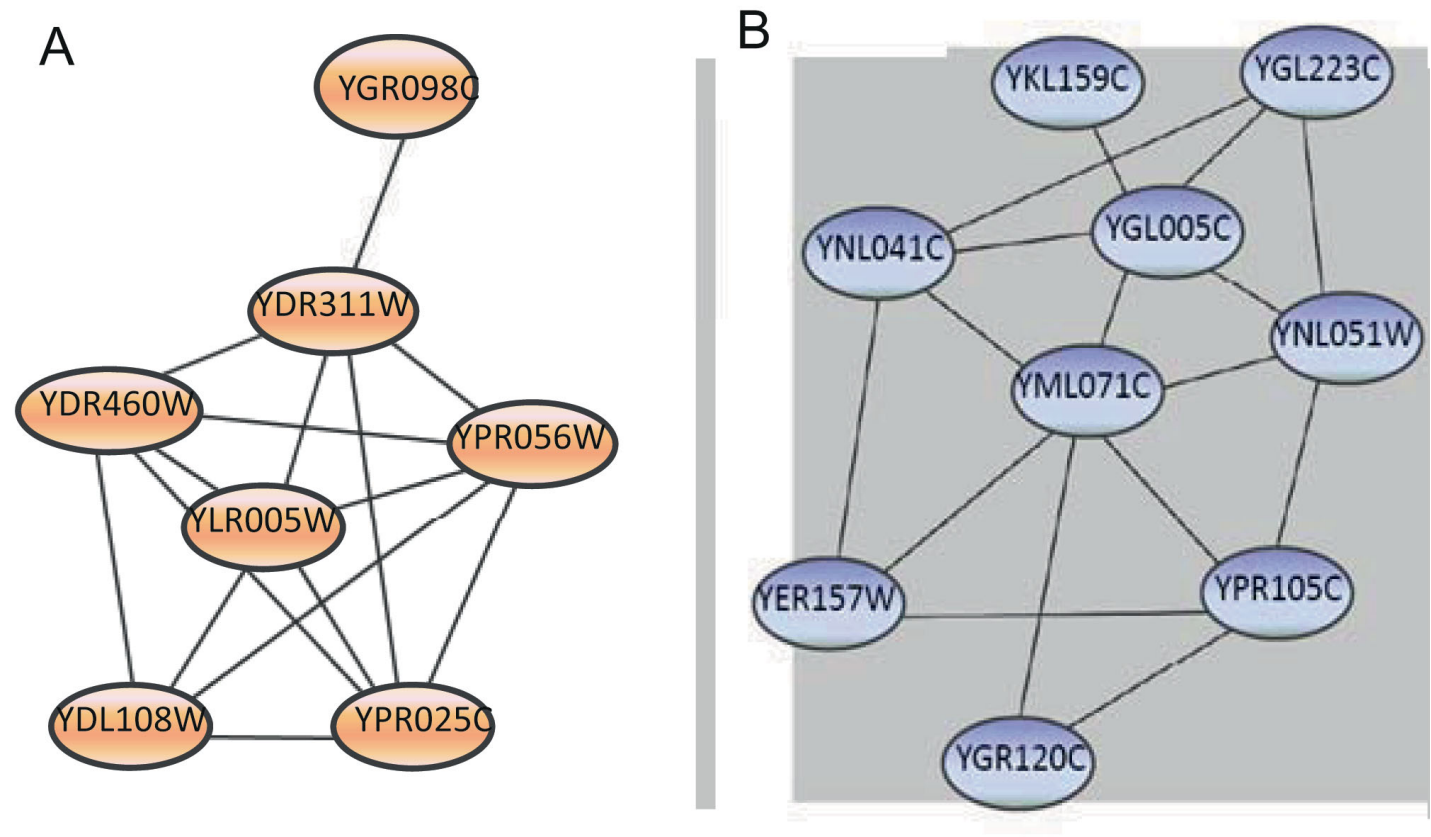
**Examples of modules which match the MIPS complexes** with great significance. (A) A seven-member module matches with the SSL2-core TFIIH complex when it is part of the nucleotide-excision repair factor 3 (NEF3) (*P_ol_* = 10^-14.^^81^). (B) A nine-member module matches with Golgi transport complex which stimulates intra-Golgi transport and is composed of eight proteins (*P_ol_* = 10^-21.^^7^).

The budding yeast *S. cerevisiae* PPI network was obtained from the DIP database (http://dip.doe-mbi.ucla.edu/dip/), which contains human-curated high-throughput and small-scale binary interactions directly observed in experiments, as well as binary interactions inferred from high-confidence protein complex data. We only considered non-self physical interactions and built the PPI network. The giant component of the PPI network is composed of 2559 proteins linked by 7031 nonredundant interactions. In order to test the ability of SpeMD to extract complexes from the interaction network and compare it with other two methods, we compared the detected modules to known complexes in yeast as annotated by the Munich Information Center for Protein Sequences (MIPS) [27] using the *P_ol_* formula. We apply the SpeMD method to the yeast PPI network to detect functional modules. Totally, we obtain 279 protein modules of sizes from 4 to 38 with λ = 0.6 (To extract statistically and biologically significant modules, we remove 48 modules with size ≤ 3). In Table 1, we show detailed information of 15 detected modules and their matching with experimentally determined protein complexes that were catalogued in MIPS database. Figure 3 presents two such modules. For example, the second one is a nine-member module which matches with Golgi transport complex for stimulating intra-Golgi transport with *P_ol_* = 10^−21.7^.

**Table 1:** Illustration of detected modules.

Module	#Module	Complex	#Complex	#Overlap	— log10(*P_o_ I*)
3	11	510.70.20	12	8	17.74
4	5	410.33	4	4	11.55
10	10	440.14.10	10	8	19.16
20	6	510.190.50	10	5	11.36
26	12	270.20.30	9	9	22.56
28	7	510.100	9	6	14.81
39	6	510.150	5	5	14.06
56	29	500.60.20	31	20	34.38
94	26	510.40.20	21	19	39.06
100	9	260.20.40	8	8	21.7
104	6	410.40.30	5	5	14.06
127	14	510.10	14	10	21.51
131	34	360.10.10	15	15	29.38
148	19	510.190.10.20.1	16	12	23.98
174	23	510.40.10	13	10	18.77

Illustration of 15 detected modules and their matching with experimentally determined protein complexes that were catalogued in MIPS database.

### Comparison with MCL and MQ

There has been many methods for detecting network modules. The comparison of all the methods is not an easy task. Here, we attempt to compare the MD (SpeMD) with two types of classical methods: MQ and MCL. Just as we have mentioned, the modularity (*Q*) maximization based module-detection method has been comprehensively applied in many fields including analysis of biological networks. Another method is the Markov Cluster algorithm (MCL) which was developed by van Dongen [29]. The method simulates a flow on the network by calculating successive powers of the network adjacency matrix. In each iteration, an inflation step is applied to enhance the contrast between regions of strong or weak flow in the network. The process converges towards a partition of the network, with a set of high-flow regions separated by boundaries with no flow. The value of the *inflation parameter* strongly influences the the size and number of the detected modules. In a recent evaluation study [28], the algorithm was found to be superior to several representative graph clustering algorithms including MCODE [9], RNSC [11] and SPC [8] for the prediction of protein complexes.

**Figure 4:**
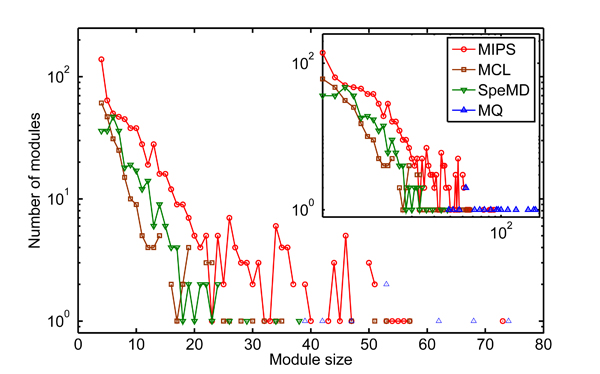
**Module size distribution** of different methods and MIPS protein complex with size > 3.

**Figure 5:**
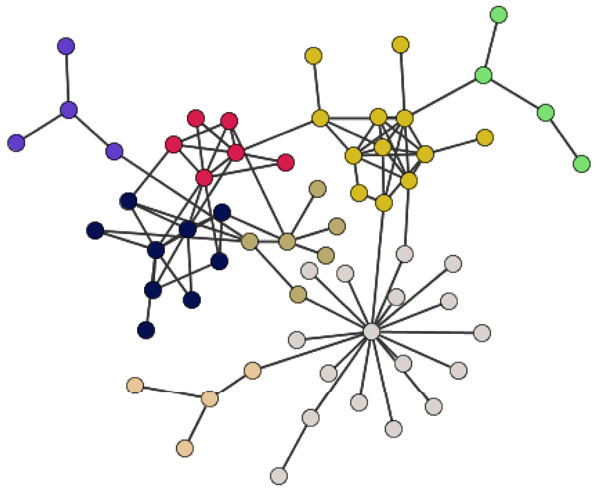
**The modular organization of a MQ module.** A MQ module with 62 proteins. We can clearly see this subnetwork show clear modular organization. The proteins with different colors are membership of eight SpeMD modules which correspond to more specific biological relevance (results not shown).

The module size distribution of detected modules for each method on the PPI network have been shown in Figure 4. The SpeMD and MCL identify 279 and 242 modules respectively without extremely large clusters (λ = 0.6 for SpeMD and inflation parameter = 1.7 for MCL). The major trend generated by MD and MCL are both similar to that of the complexes in MIPS database, which suggest the definition of modularity density is reasonable (Note that the MIPS complex is a combination of hand-curated and experimental complexes. They have some overlap, so complex curve is higher. But the trend is similar). Unfortunately, the module size distribution of MQ is very different from the previous ones. The MQ only detect 21 modules with relative large size ranging from 39 to 263. As tested on the simulated networks, the MQ method is highly limited by the resolution problem (Figure 5).

**Figure 6:**
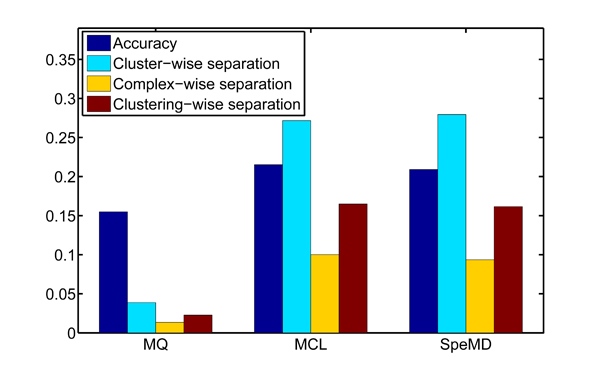
**Performance of different methods** on the PPI network. Four different measures including 'Accuracy', 'Cluster-wise separation', 'Complex-wise separation, and 'Clustering-wise separation' have been used.

As to biological significance, the accuracy and separation are used for evaluating the correspondence between complexes and modules from each method [28]. From Figure 6, we can easily see that the SpeMD and MCL have consistently better performance than MQ. This means the modularity density based partition method can produce more biologically significant modules than the modularity based method. And the new quality function may become an evaluation index of modularity organization of networks. While MCL has no such evaluation function.

## Discussion and conclusion

PPI networks are typical examples of complex biological systems that are difficult to understand from raw experimental data alone. Algorithmic and modeling progresses in the area of biomolecular networks analysis could contribute to the understanding of biological processes and organization. Many methods have been developed to organize, display and extract significant patterns in these systems [2]. 

A number of network clustering algorithms have been proposed to find modular structures in PPI networks and other biological networks. Our work is a further development along this line for dissecting biological systems. Here we introduce the quantitative measure (Modularity density *D*) for exploring modular organization of networks to the field of biomolecular networks. We suggest the SA technique to maximize it for rigorous evaluation and we propose an efficient spectral -means method in the decomposition procedure. Our comparative experiments with MCL and MQ on a yeast PPI network show that the MD (SpeMD) method can effectively detect protein interaction modules from a complex interaction network. In the current research, we use known complexes to choose the optimal λ as well as the inflation parameter for MCL algorithm. Actually, we can also adopt an intrinsic measure which compares the resulting modules against the original network to choose the most appropriate parameter in an unsupervised manner. For example, van Dongen [29] suggested the so-called *efficiency* measure to test the performance of network clustering efficiency. Therefore, the present method can be easily adapted to a fully self-contained method that doesn't rely on any known data or given parameters. The current algorithm, as most clustering methods, uncovers only disjoint modules (clusters). However, in real biological systems, proteins can be contained in more than one functional module or complex. Zhang *et al*. (2007c) has suggested to apply fuzzy c-means clustering method to a spectral space for uncovering fuzzy modules [25]. It can also be addressed using an intrinsic measure based on the original network in the same way as suggested in [30] by post-processing the modules obtained from the present algorithm.

Modularity *Q* have been extensively employed for dissecting and evaluating the modular organization of biomolecular networks [3,17-19] as well as clustering the graphic representation of gene expression profile data [31]. However, the heavy resolution limit of modularity *Q* reminds researchers to use it cautiously. And the modularity density *D* may become an alterative measure to achieve these goals.

In summary, our method is very effective for uncovering modular organization in biomolecular networks. It provides an objective approach to explore the organization and interactions of biological processes. With the increasing amount of biological ‘interaction' data available, MD (SpeMD) can facilitate the construction of a more complete view of the composition and interconnection of functional modules and the understanding of the organization of the whole cell at system level. We plan to automate this algorithm to compute functional modules for a large number of biological networks. We hope that related studies will benefit from the present method coupled with the modularity density *D* (*D*_λ_).

## Competing interests

The authors declare that they have no competing interests.

## Authors' contributions

SZ designed the study. SZ and XMN implemented the method, performed the experiments and analyzed the data. CD and XSZ contributed to discussions on the method. SZ, XMN, CD and XSZ wrote the manuscript.
